# Towards a new pseudo-quantitative approach to evaluate the ionization response of nitrogen compounds in complex matrices

**DOI:** 10.1038/s41598-021-85854-7

**Published:** 2021-03-19

**Authors:** Julie Guillemant, Marion Lacoue-Nègre, Alexandra Berlioz-Barbier, Florian Albrieux, Luis P. de Oliveira, Jean-François Joly, Ludovic Duponchel

**Affiliations:** 1 IFP Energies Nouvelles, Rond-Point de L’échangeur de Solaize, BP 3, 69360 Solaize, France; 2 CNRS, UMR 8516 - LASIRE – Laboratoire avancé de spectroscopie pour les interactions, la réactivité et l’environnement, Univ. Lille, 59000 Lille, France

**Keywords:** Analytical chemistry, Energy

## Abstract

Ultra high-resolution mass spectrometry (FT-ICR MS) coupled to electrospray ionization (ESI) provides unprecedented molecular characterization of complex matrices such as petroleum products. However, ESI faces major ionization competition phenomena that prevent the absolute quantification of the compounds of interest. On the other hand, comprehensive two-dimensional gas chromatography (GC × GC) coupled to specific detectors (HRMS or NCD) is able to quantify the main families identified in these complex matrices. In this paper, this innovative dual approach has been used to evaluate the ionization response of nitrogen compounds in gas oils as a case study. To this extent, a large gas oil dataset has been analyzed by GC × GC/HRMS, GC × GC-NCD and ESI(+/−)-FT-ICR MS. Then, the concentrations obtained from GC × GC-NCD have been compared to those obtained from FT-ICR MS hence proving that strong ionization competitions are taking place and also depending on the origin of the sample. Finally, multilinear regressions (MLR) have been used to quantitatively predict nitrogen families from FT-ICR MS measurements as well as start rationalizing the ionization competition phenomena taking place between them in different types of gas oils.

## Introduction

The most used method nowadays to quantify species of interest in complex matrices such as petroleum products is comprehensive two-dimensional gas chromatography (GC × GC) coupled to either high resolution mass spectrometry (HRMS)^1, 2^ or specific detectors such as nitrogen chemiluminescent detector (NCD) for the absolute quantification of nitrogen compounds^3–5^ or sulfur chemiluminescent detector (SCD) for the absolute quantification of sulfur compounds^6^. However, information obtained with these methods is sometimes not sufficient to explain the molecular differences between two samples. On the other hand, ultra-high resolution mass spectrometry (FT-ICR MS) provides an unsurpassed level of detail and usually more than 10 times faster than GC × GC-NCD analysis hence improving analysis efficiency. FT-ICR MS is often coupled to electrospray ionization (ESI) to characterize polar compounds within complex mixtures but ESI undergoes important and complex ionization competition phenomena between the compounds of interest as well as possible different ionization responses depending (1) on the samples (matrix effect), (2) on the ionization efficiency of the compounds themselves and (3) the used ionization conditions (solvents, additives, dilution…)^7–9^. On the other hand, multiple linear regression (MLR) can be used to quantify some complex analytes while considering the matrix effect related to the analytical method used that could prevent the quantification^10, 11^. As an example, Cakara et al.^12^ proposed an interesting MLR approach to quantify the exact amounts of Mo, Si and B in different alloys from ICP-MS measurements. The MLR models included the possible impurities in the alloys as well as isobars species which strongly helped to correct the matrix effects observed when performing ICP-MS.

In this paper, a dual approach combining both GC × GC and MLR has been used to evaluate the ionization responses of nitrogen compounds found in gas oils. The hydrotreatment (HDT) process aims at removing impurities such as sulfur, nitrogen or metals from gas oils cuts so that they can respect the legal specifications. As these are getting more and more stringent, the hydrotreatment process needs to be more and more efficient and despite their relatively low amount among the gas oils, the nitrogen compounds are of high concern for refiners^13–16^. Neutral nitrogen compounds are refractory while the basic ones deactivate the catalysts used and compete with sulfur compounds for their removal while the sulfur content should be lower than 10 ppm^17, 18^. The total nitrogen content remaining in the hydrotreated samples is a key information for assessing the efficiency of the hydrotreatment. Unfortunately, it does not provide sufficient information to understand the mechanisms involved in this process^19^. In order to remove as much nitrogen as possible, one needs to get access to a detailed characterization of nitrogen compounds allowing to design targeted catalysts or to choose wisely the hydrotreatment conditions to favor nitrogen removal. A detailed quantitative characterization of both feeds (gas oils processed by hydrotreatment) and effluents (gas oils obtained after hydrotreatment) would be helpful to help understanding these mechanisms and thus improving the overall efficiency of hydrotreatment^20, 21^.

Some pseudo-quantitative approaches for petroleum matrices have already been reported in the literature using mainly GC × GC analysis to evaluate the potential of FT-ICR MS as a pseudo-quantitative tool but mainly for sulfur compounds and without considering both basic and neutral nitrogen compounds^22–24^. Besides, these studies are based on a small number of gas oils which is not sufficient to identify a possible matrix effect from the gas oils and they did not consider simultaneously the impact of the other ionized families over the ionization response of a given family.

This paper aims at assessing the efficiency of a combined GC × GC and MLR approach to evaluate the ionization responses of nitrogen compounds in complex matrices, here gas oils, during ESI(+/−)-FT-ICR MS analysis. As a first step, GC × GC/HRMS was used to verify the assignments of the nitrogen compounds eluted by GC × GC-NCD and to obtain blobs depending on the Double Bond Equivalents (DBE) of the compounds. A total of 21 gas oils was then analyzed by GC × GC-NCD and ESI(+/−)-FT-ICR MS. Then, MLR modeling was used to predict the GC × GC-NCD concentrations from FT-ICR MS measurements for the different nitrogen families in a given ionization mode. The obtained regression coefficients gave clues about the observed ionization competitions between the nitrogen compounds.

## Results

### Introduction of GC × GC-NCD blobs using GC × GC/HRMS

Usually, the GC × GC-NCD blobs are identified as a function of the nitrogen family (indoles, carbazoles, TetraHydroQuinolines [THQ], anilines…) while the identification of the compounds and their derivatives when performing FT-ICR MS measurements is based on DBE and their basic/neutral character. The basicity of the nitrogen compounds can be linked to six-membered nitrogen heterocycles on which the free pair of electron of the nitrogen atom is not involved in the π-electron system which gives a basic character to these types of molecules, which is the case of molecules such as THQ, anilines or again acridines. On the opposite, when five-membered heterocycles are considered, the free electron pair of the nitrogen atom is conjugated within the π-electron cloud of the ring which suppresses almost all the basicity of the compounds which can be considered as neutral such as indoles or carbazoles. As an example, three different blobs corresponding to tetrahydroquinolines, anilines and pyridines can be observed while only one family is considered for FT-ICR MS analysis as seen in Figure S1 in Supporting Information. Moreover, the tetrahydrocarbazoles (THC) family is identified in a single blob while these compounds are identified in the indoles family for FT-ICR MS measurements as their DBE equal to 7 is contained between 6 and 8. Thus, in order to be able to compare the two methods, it was therefore necessary in a first step to create new blobs based on the DBE values of the compounds rather than the nitrogen families.

**Figure 1:**
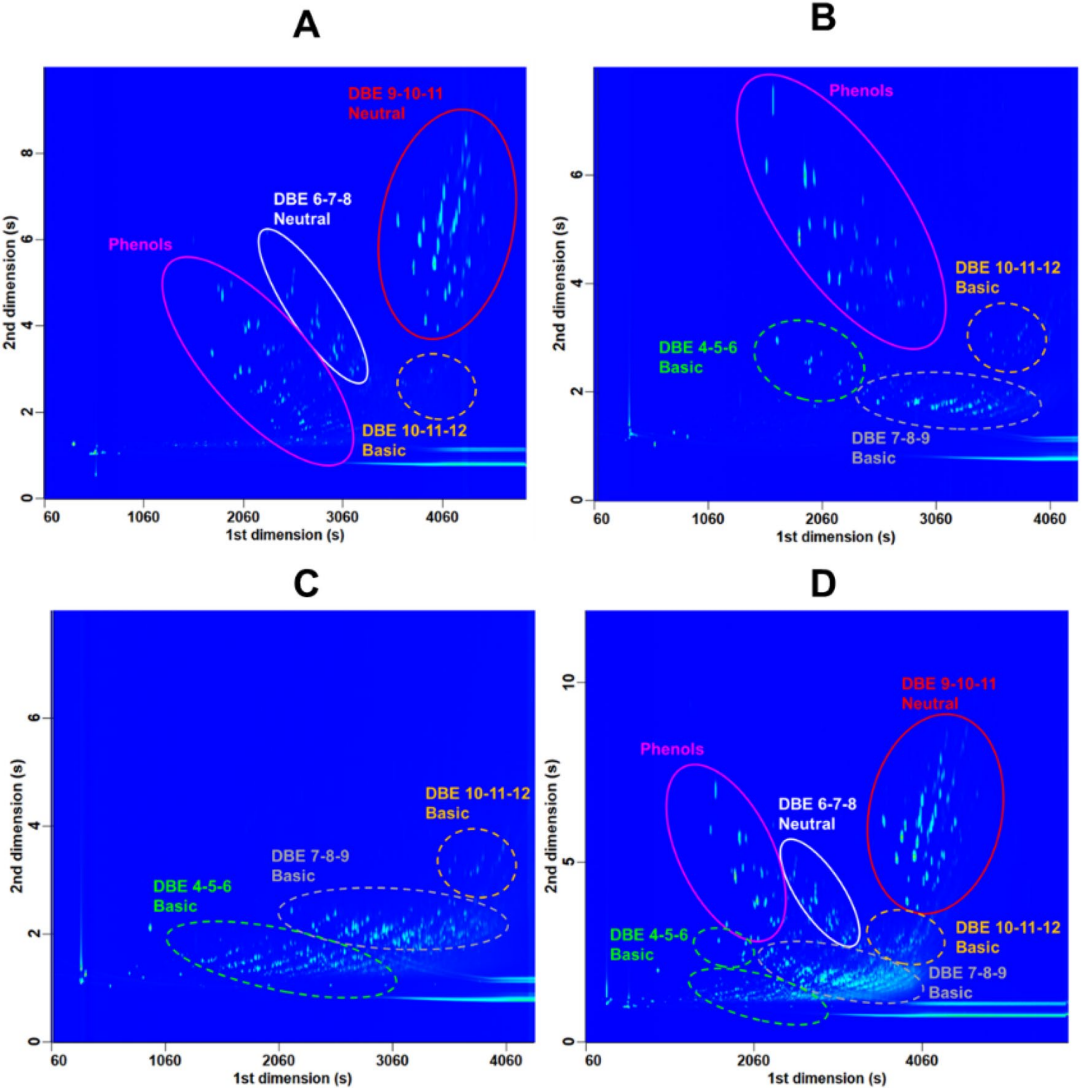
GC × GC/HRMS chromatograms obtained for the fraction F3 (**A**), F4 (**B**), F5 (**C**) and the mixed solution F3F4F5 (**D**) considering the GO 10 sample. The chromatograms were obtained using ChromaTOF software version 5.2 (LECO, St Joseph, MI, USA, https://www.leco.com/product/chromatof-software).

To obtain these blobs, GC × GC/HRMS analysis was performed but it is not very sensitive to nitrogen compounds when the whole gas oil sample is analyzed as only hydrocarbons are mostly detected. The gas oil samples were then pre-fractionated to extract the neutral and basic nitrogen compounds from the hydrocarbon matrix prior to GC × GC/HRMS analysis^25^. The first two fractions contained the hydrocarbons. Three fractions containing the nitrogen compounds were obtained: one with the neutral nitrogen compounds (F3), one with some intermediate basic compounds (F4) and one with most of the basic compounds (F5). These fractions have been volume to volume mixed into a single solution called F3F4F5 to reconstruct the gas oil without the hydrocarbon matrix. An example of GC × GC/HRMS chromatogram obtained from the fractions of the GO 10 sample as well as their mixed solution F3F4F5 is shown in Fig. 1.

To facilitate the identification, the modulation periods and temperature gradients have been optimized for each fraction to maximize the chromatographic space occupancy of the fractions. The mixed solutions F3F4F5 were analyzed strictly in the same conditions as GC × GC-NCD for comparisons. The identification of the molecular formula of the compounds was possible thanks to the use of HRMS and some blobs were later created based on DBE values of these compounds.

Neutral nitrogen compounds are identified into blobs shaped as plain ellipses in Fig. 1 while basic ones are identified with dotted line ellipses. The fraction F3 (Fig. 1A) mainly contains neutral nitrogen compounds with DBE values equal to 9, 10 and 11 that correspond to carbazoles family. It also contains other neutral nitrogen compounds with DBE values equal to 6, 7 and 8 that correspond to tetrahydrocarbazoles and indoles families. Some phenolic compounds are also found as well as very aromatic basic compounds in very low proportion with DBE values between 10 and 12 that correspond to benzoquinolines or acridines derivatives. The fraction F4 (Fig. 1B) contains poorly aromatic basic compounds with DBE values equal to 4, 5 and 6 that correspond mainly to anilines derivatives. Intermediate basic compounds with DBE values equal to 7, 8 and 9 are also observed which correspond to quinolines derivatives. We also observe very aromatic compounds with DBE values between 10 and 12 (acridines/benzoquinolines). Some phenols derivatives are again identified. Finally, the F5 fraction (Fig. 1C) only contains basic compounds throughout the whole DBE range (i.e. from 4 to 12).

One can note that the type of compounds with DBE values equal to 4, 5 and 6 and eluted in the fractions F4 and F5 are different as their retention are different. Indeed, compounds with DBE values equal to 4, 5 and 6 identified in the fraction F5 are mainly tetrahydroquinolines and pyridines derivatives while the anilines are identified in the fraction F4. As these compounds are simultaneously identified in one family by FT-ICR MS, the sum from both blobs will be considered to obtain the concentration of the THQ Anilines Pyridines family. The mixed solution F3F4F5 contains all blobs previously identified in the fractions which confirms the reconstruction of the nitrogen gas oil matrix without hydrocarbons (Fig. 1D).

**Figure 2:**
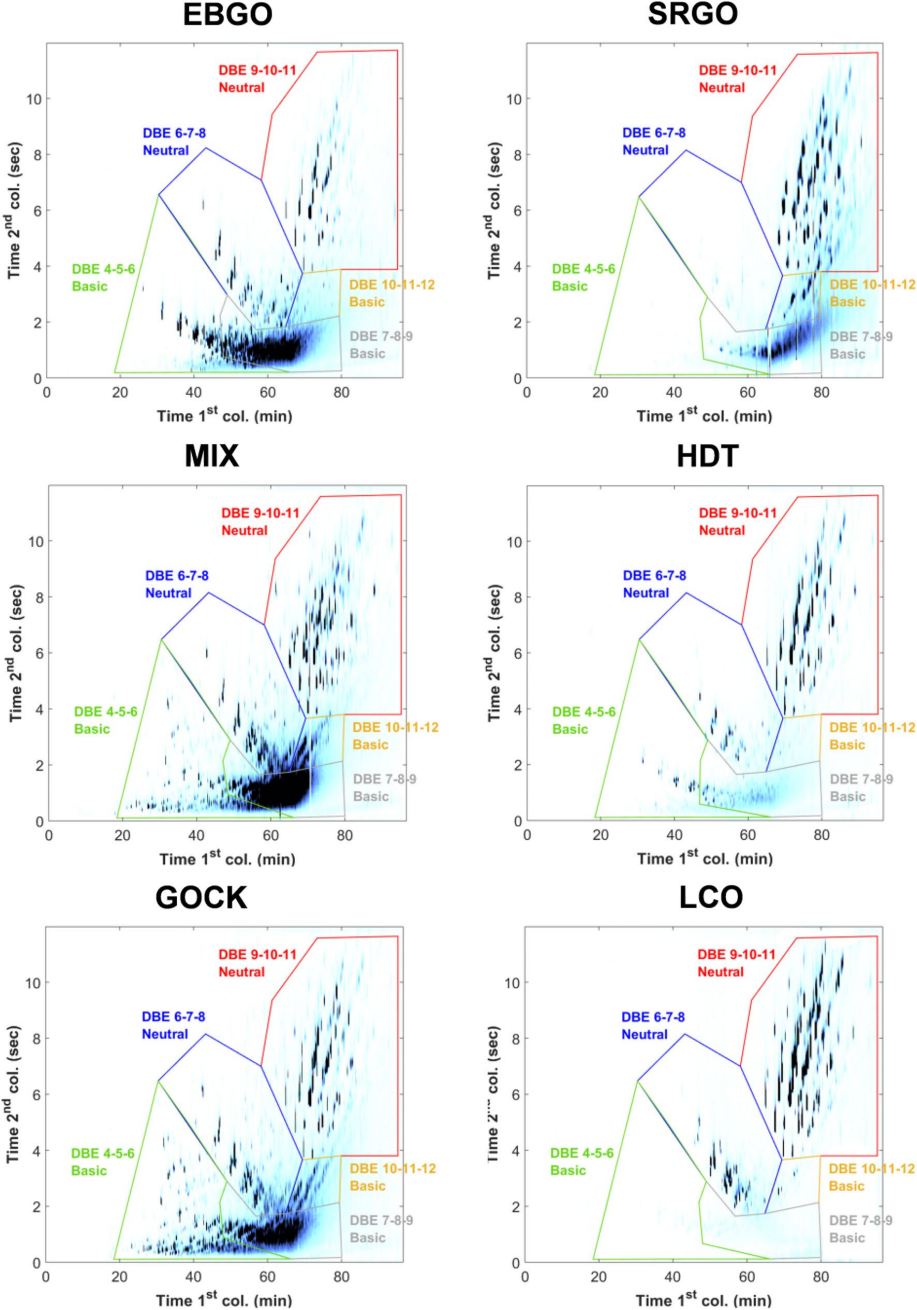
GC × GC-NCD chromatograms obtained for the different types of gas oils considered. The chromatograms were obtained using a home-made software 2D Chrom.

The previously defined blobs were then used for the analysis of entire gas oils data base using GC × GC-NCD as the NCD detector is only specific to nitrogen compounds. Among this gas oil database, we can mention different industrial types of gas oils such as the Straight Run Gas Oils (SRGO), the Light Cycle Oils (LCO), the Coker Gas Oils (GOCK), the Gas Oils from Ebullating Bed hydroprocessing reactor (EBGO), the Gas Oils from Fixed Bed hydroprocessing reactor (FBGO) or again the Hydrotreated gas oils (HDT). Some examples of GC × GC-NCD chromatograms obtained for a EBGO sample (GO 12), a SRGO sample (GO 3), a LCO sample (GO 5), a MIX sample (GO 21), a HDT sample (GO 18) and a GOCK sample (GO 10) are shown in Fig. 2. The repartition of nitrogen compounds within the different blobs is different depending on the gas oil considered. The distributions of the GOCK and EBGO samples are quite similar and reveal an even distribution of neutral and basic nitrogen compounds that are both poorly aromatic (basic DBE 4–5–6 and neutral DBE 6–7–8) and quite aromatic (neutral DBE 9–10–11 and basic DBE 10–11–12). The MIX sample is a mixed blend between 67% GOCK and 33% LCO samples so its composition is very similar to those of GOCK sample. In comparison, the LCO sample mainly contains neutral nitrogen compounds as the basic nitrogen content in these samples is very low (see Table 1) but some very aromatic basic compounds are identified in low proportion. The SRGO composition is quite atypical: the identification blobs do not seem to fit this type of gas oil as a shift of the blobs is needed due to a modification of the retention time of the compounds.

**Table 1:** Macroscopic properties of gas oil samples used in this study.

Sample	Type	Total nitrogen (ppm) Ref. method: ASTM D4629	Basic nitrogen (ppm) Ref. method: ASTM D2896	Density at 15 °C (g/cm^3^) Ref. method: ASTM D4052
GO 1	SRGO	115	47	0.8541
GO 2	SRGO	254	100	0.8665
GO 3	SRGO	350	129	0.8878
GO 4	LCO	928	91	0.9130
GO 5	LCO	1170	49	0.9413
GO 6	LCO	496	141	0.9035
GO 7	LCO	925	98	0.9310
GO 8	GOCK	893	404	0.8501
GO 9	GOCK	838	390	0.8581
GO 10	GOCK	1200	449	0.8640
GO 11	GOCK	1260	569	0.8813
GO 12	EBGO	1719	855	0.8712
GO 13	FBGO	195	121	0.8522
GO 14	HDT	93	14	0.8585
GO 15	HDT	140	23	0.8591
GO 16	HDT	205	38	0.8617
GO 17	HDT	464	107	0.8678
GO 18	HDT	723	330	0.8691
GO 19	MIX	586	122	0.8828
GO 20	MIX	380	63	0.8708
GO 21	MIX	988	436	0.8576

The neutral nitrogen content is calculated by deducting the basic nitrogen from the total nitrogen content. The ASTM standard methods used for analysis are mentioned for each property.

*SRGO *straight run gas oil, *LCO* light cycle oil, *GOCK *coker gas oil, *EBGO* gas oil from ebullating bed hydroprocessing reactor, *FBGO* gas oil from fixed bed hydroprocessing reactor, *MIX* blended gas oil, *HDT *hydrotreated.

This has been observed for the three SRGO samples so it has been assumed that this might be due to the hydrocarbon matrix of the SRGO samples. Thus, a mixed solution of the F3F4F5 fractions obtained for the GO 3 sample (SRGO) that do not contain hydrocarbons has also been analyzed by GC × GC-NCD and the chromatogram has been compared to the one obtained for the whole gas oil. No significant difference has been spotted and the shift was still observed so (1) the hydrocarbon matrix was excluded from possible explanations of the shift and (2) no gain is observed between the GC × GC-NCD analyses of the whole and the pre-fractionated gas oils. Thus, it might be due to the nitrogen intrinsic matrix of the SRGO samples. Finally, the HDT sample mainly contains neutral nitrogen compounds with DBE values equal to 9, 10 and 11 which is consistent with the well-known refractory behavior of carbazole compounds. In lower proportion, poorly aromatic basic compounds (DBE 4–6) are also identified. Generally speaking, the GC × GC-NCD chromatograms allow extracting global features of the different types of gas oils but the detail level is by far lower than those obtained by FT-ICR MS analysis (DBE, number of carbon atoms…) described in previous work4. Thus, the evaluation of FT-ICR MS measurements as a pseudo-quantitative tool has been assessed in the next sections.

### Comparison between GC × GC-NCD and FT-ICR MS

An example of mass spectra and corresponding DBE = f(#C) plots for the four main types of gas oils are available in Figure S4 for ESI(+) mode and in Figure S5 in ESI(+) mode in Supporting Information. As a first step, the GC × GC-NCD and FT-ICR MS pseudo-concentrations obtained for the 21 gas oils have been plotted for each nitrogen family. As a reminder, the pseudo-concentrations were then obtained by multiplying the relative intensity times the amount of elemental neutral nitrogen (for ESI(-) data) or basic nitrogen (for ESI(+) data).

**Figure 3:**
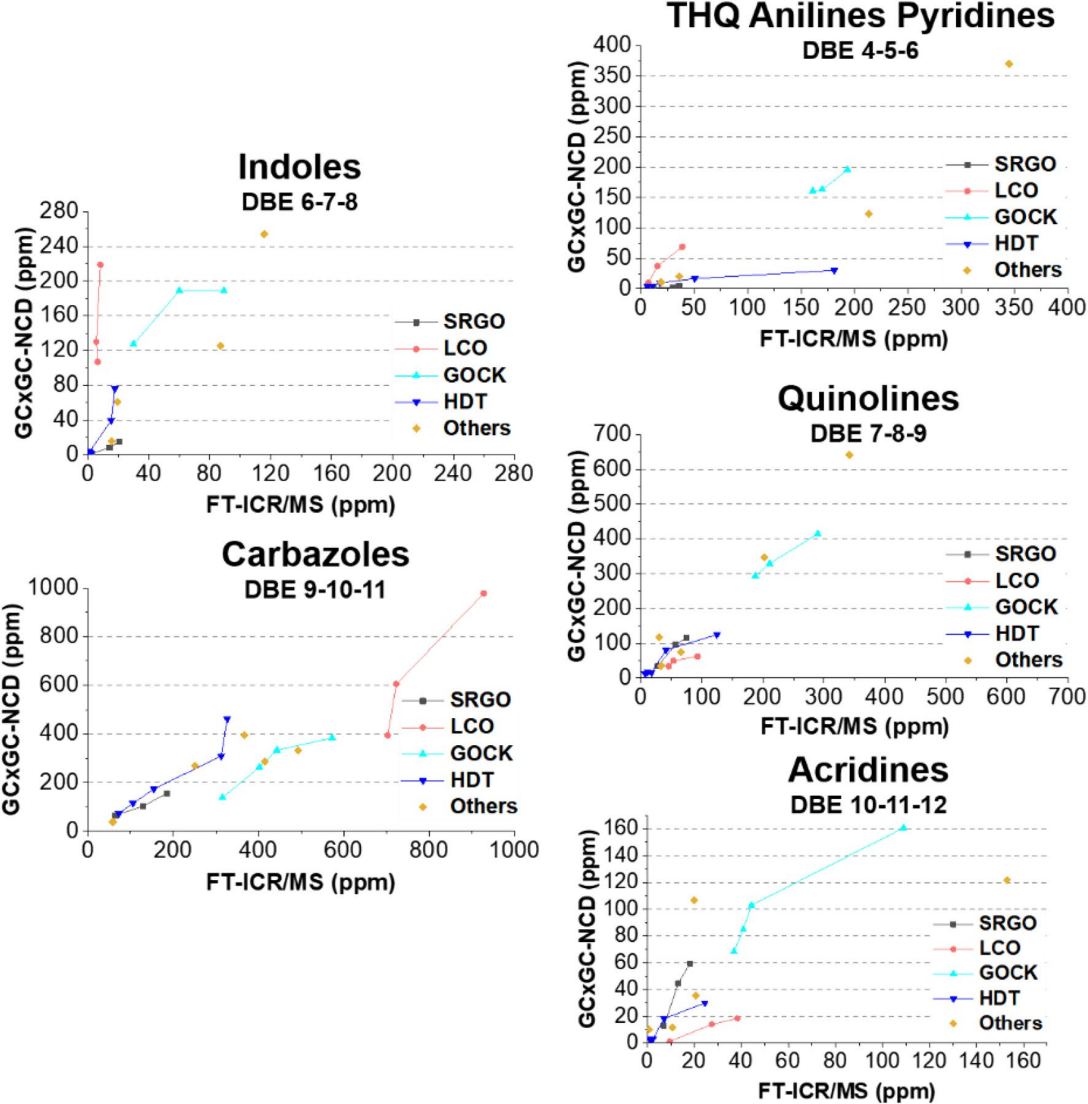
Comparison of the pseudo-concentrations obtained by GC × GC-NCD as a function of the pseudo-concentrations obtained by FT-ICR MS for the different nitrogen families.

To facilitate the reading, the most abundant compounds in the DBE families have been used such as indoles for the family containing the neutral DBE 6–7–8 or Acridines for the family containing the basic DBE 10–11–12. Thus, it has to be mentioned that these so-called families include the corresponding DBE derivatives of the compounds than can only be clearly assigned by families using GC × GC as an access to the structure of the compounds is achieved. Moreover, it could also been mentioned that as the FT-ICR/MS analyses are not able to provide further access to the structure, the impact of the molecule structure over the ionization efficiency has not been assessed but is of major importance. These comparisons are available in Fig. 3 where the different points have been colored depending on the type of gas oil.

It is quite obvious that whichever family considered, no linear trend is observed between both techniques when all samples are compared. However, if we consider the points corresponding to the same type of gas oil, some local trends could be identified. This shows that the ionization response of the nitrogen compounds in the gas oils is different depending on the type of gas oil and confirms the strong need of a large and very diverse gas oil database to perform this kind of study. It has been mentioned in the introduction section that the different phenomenon that could impact the quantification of these compounds in ESI are (1) the matrix effect linked to the nature of the samples, (2) the ionization efficiency of the compounds and (3) the ionization conditions used to perform the analyses. In this case, as the ionization conditions (3) were strictly the same for all samples, the differences observed for the different types of gas oils can not be correlated to them. As regards the matrix effect (2), even though hydrocarbons are not ionized when performing ESI, a possible hydrocarbon matrix could affect the ionization of nitrogen compounds. To evaluate this, the mixed solutions F3F4F5 from pre-fractionated samples GO 3, GO 10, GO 12 and GO 17 have been analyzed by ESI(+/−)-FT-ICR MS. The evolutions of the relative intensity as a function of the DBE values have been compared to those obtained when analyzing the whole gas oils. No significant changes were observed neither in ESI(+) or ESI(−) modes which means that there was no hydrocarbon matrix effect whichever gas oil sample considered (see Figures S2 and S3 in Supporting Information). Then, it has been assumed that the different nitrogen ionization responses are due to ionization competitions between nitrogen compounds linked to the different ionization efficiencies from the compounds of interest (3). As a consequence, a strong ionization suppression effect might be induced by the intrinsic nitrogen gas oil matrix and new descriptors need to be introduced to evaluate these ionization efficiencies or possible ionization competitions for each nitrogen family.

### MLR modeling

**Figure 4:**
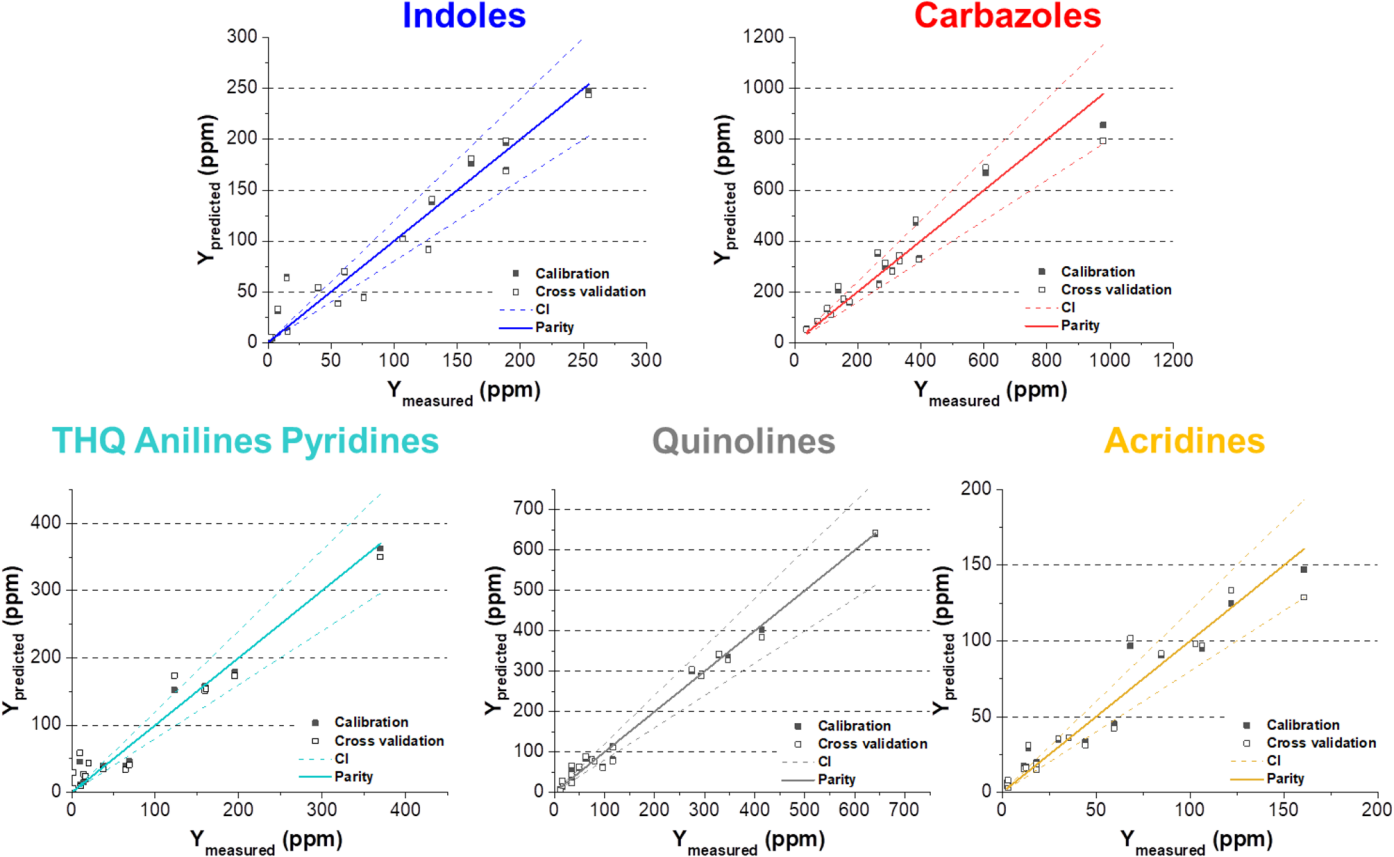
Parity diagrams showing the predicted Y value as a function of the measured Y value obtained from MLR models.

As a second step, a multiple linear regression approach was used to consider simultaneously the FT-ICR MS measurements from several nitrogen families assigned within a given ionization mode and thus identify the deviations observed by FT-ICR MS. In this case, the predicted GC × GC-NCD concentrations obtained from the MLR models were compared to the measured GC × GC-NCD concentrations. It should be mentioned that the FT-ICR MS measurements considered here correspond to the mean of six independent measurements for a given sample while no replicates have been performed when using GC × GC-NCD. Thus, some samples whose resulting GC × GC-NCD analysis were atypical (i.e. not similar to those obtained from the same gas oil class) have been removed from calibration to avoid including more analytical variance. All variables were auto-scaled. Due to the low number of samples available, all samples were used for calibration and a cross-validation procedure was used to estimate the models accuracies which is a standard statistical practice under these particular conditions. The characteristics of the developed MLR models for all families are summarized in Table S1 in Supporting Information. The evolution of the predicted concentration as a function of the measured one for all gas oil samples in calibration and cross validation for all nitrogen families are shown in Fig. 4. On each graph, the first bisector, also called parity line, is plotted. A perfect model will have all samples along this line. Upper and lower bounds of the confidence interval are also given for each graph using dotted lines. It is calculated considering an assumed 10% relative error of the reference GC × GC-NCD method. Globally, the very low FT-ICR/MS concentrations are not very well predicted by the model because the global model is not able to correct such high difference between GC × GC-NCD and FT-ICR/MS values.

GC × GC-NCD only provides quantification of purely nitrogen containing compounds and is not able to quantify oxygen, sulfur of heteroatomic species that obviously also impact the ionization of nitrogen compounds in both positive or negative electrospray modes. Thus, only the impact of the nitrogen families (N1 class) is discussed in the following sections but the impact of these other families should not be in any case neglected.

#### Indoles family and their derivatives

The GO 1, GO 21 and GO 7 samples were removed from the MLR model developed for indoles family as very atypical FT-ICR MS values were observed for these samples. Predicted concentrations of indoles versus measured ones are presented in Fig. 4. Globally, most of the points are located quite near the parity line within the confidence intervals even if the model does not seem to fit perfectly the very low concentrations (< 20 ppm). The calibration and cross-validation coefficients of determination (R^2^_calibration_ and R^2^_cross validation_) are respectively equal to 0.94 and 0.93 which are good figures of merit. No significant biases are observed neither for calibration or cross-validation. A satisfactory prediction is observed on all samples with also rather good errors: the RMSEC and RMSECV values are respectively equal to 19 ppm and 20 ppm which is satisfying. The coefficients of regression for the predictive model of a given neutral family are given in the Eq. (1):

1 $$\left[ {{\text{GC}} \times {\text{GC - NCD}}} \right]_{{{\text{predicted}}}} =\upbeta _{{{\text{Indoles}}}} \times \, \left[ {{\text{FT - ICR}}\;{\text{MS}}} \right]_{{{\text{Indoles}}}} + \upbeta _{{{\text{Carbazoles}}}} \times \left[ {{\text{FT - ICR}}\;{\text{MS}}} \right]_{{{\text{Carbazoles}}}}$$

With here β_Indoles_ =  + 0.7278 and β_Carbazoles_ =  + 0.4745. Since all variables have been centered and reduced prior the model development, we can therefore directly use the absolute values of the beta coefficients but also their signs to develop an interpretation of the constructed models. First, the absolute values will indicate which variables are the most influential for concentration prediction. For the indoles model, both FT-ICR MS pseudo-concentrations influence the model. Moreover, the sign of the coefficient allows evaluating if it corresponds to a corrective factor (negative sign) or a compensative factor ( +) to correct either the over or under estimation of FT-ICR MS measurements.

In this case, the contribution of the carbazoles pseudo-concentrations indicates that the indoles pseudo-concentrations are not sufficient alone to efficiently predict the GC × GC-NCD concentration and that their ionization is impacted by carbazoles compounds. A positive coefficient is linked to FT-ICR MS measurements so an under estimation of the actual GC × GC-NCD value is observed when performing FT-ICR MS. It can be assumed that there are some strong ionization competition phenomenon occurring between indoles and carbazoles. Indeed, the pKa of carbazoles compounds (− 6) is lower than the one of indoles compounds (− 2.4). Considering the obtained working pH with the addition of 0.15% of ammonium hydroxide, carbazoles compounds and their derivatives should be fully deprotonated in solution. ESI(−) ionization mechanism being based on the deprotonation of the compounds, carbazoles are more likely to be more easily ionized than indoles as they are already deprotonated in solution. As a consequence, the indoles are probably under ionized by ESI(-)-FT-ICR MS and this is consistent with the positive contribution of this variable. The positive sign assigned to carbazoles implies that the carbazoles act as a compensative factor and that the under ionization of indoles is enhanced within gas oils that have high contents in carbazoles. This is actually the case as FT-ICR MS pseudo-concentrations of indoles compounds are strongly underestimated within GOCK and LCO samples as these samples have high contents in carbazoles (see Fig. 3). As benzocarbazoles compounds were also identified by FT-ICR MS analysis (but not detected using GC × GC-NCD), a second model was developed including the pseudo-concentrations obtained for this family (RMSEC and RMSECV equal to 19 and 21 ppm respectively). The corresponding equation is given in the Eq. (2):

2 $$\begin{aligned} \left[ {{\text{GC}} \times {\text{GC - NCD}}} \right]_{{{\text{predicted}}}} & =\upbeta _{{{\text{Indoles}}}} \times \, \left[ {{\text{FT - ICR}}\;{\text{MS}}} \right]_{{{\text{Indoles}}}} + \upbeta _{{{\text{Carbazoles}}}} \times \left[ {{\text{FT - ICR}}\;{\text{MS}}} \right]_{{{\text{Carbazoles}}}} \\ & \quad +\upbeta _{{{\text{Benzocarbazoles}}}} \times \, \left[ {{\text{FT - ICR}}\;{\text{MS}}} \right]_{{{\text{Benzocarbazoles}}}} \\ \end{aligned}$$

With here β_Indoles_ =  + 0.7065, β_Carbazoles_ =  + 0.4372 and β_Benzocarbazoles_ =  + 0.0518. As the coefficient linked to benzocarbazoles is very small, the contribution of this family in the under-ionization of the indoles compounds was assumed to be non-significant. Thus, the original model was kept (Eq. 1).

When comparing with the GC × GC-NCD and FT-ICR MS straight comparison in Fig. 3, it is obvious that including the carbazoles FT-ICR MS pseudo-concentrations into the last multivariate model significantly improves its accuracy. More importantly, it was the only way to create a link that was impossible to find only using FT-ICR MS and GC × GC-NCD analysis. Thus, it seems that the MLR model is able to flatten the intrinsic characteristics of each type of gas oil to obtain a universal response whichever gas oil considered.

#### Carbazoles family and their derivatives

The samples GO 4, GO 6 and GO 18 have been excluded from the calibration data set to obtain a more realistic model. Most of the predicted points are again found within the confidence intervals and a linear trend is observed (see Fig. 4). Calibration and cross-validation biases are quite satisfying as well as corresponding coefficients of determination (R^2^cal = 0.932, R^2^cross-val. = 0.898). The RMSEC (57 ppm) and RMSECV (70 pm) values are higher compared to the other models due to the extended concentration range (0–1000 ppm). The coefficients related to the model are the following ones: β_Indoles_ = − 0.204 and β_Carbazoles_ =  + 0.9647. Both pseudo-concentrations influence the model but the carbazoles pseudo-concentrations are predominant. The regression coefficient related to carbazoles FT-ICR MS measurements is a little under 1 indicating a small underestimation of FT-ICR MS values compared to GC × GC-NCD values for this family. On the opposite, the sign associated to indoles pseudo-concentrations is negative. The carbazoles would be globally underestimated when performing FT-ICR MS measurements but would be slightly overestimated within samples that have low indoles contents (such as LCO samples) due to the assumed ionization competitions between both families.

Another model (RMSEC and RMSECV equal to 57 and 75 ppm) including the benzocarbazoles pseudo-concentrations has been built as presented in Eq. (2) with the following coefficients: β_Indoles_ =  ± 0.2252, β_Carbazoles_ =  + 0.9124 and β_Benzocarbazoles_ =  + 0.0676. As the coefficient linked to benzocarbazoles is again small, it was considered as non-significant and the initial model was kept.

#### THQ anilines pyridines family and their derivatives

Within the MLR model obtained for the THQ anilines pyridines family, the gas oils GO 6, GO 11 and GO 18 have been excluded from the calibration data set as they behave as outliers for this model. The evolution of the predicted concentration as a function of the measured concentration in shown in Fig. 4. Most of the points are contained within the confidence intervals except some low concentration points. Calibration and cross-validation coefficients of determination are respectively equal to 0.965 and 0.938 which is very satisfying. Small calibration and cross-validation biases are also observed. Quite low values of RMSEC (18 ppm) and RMSECV (23 ppm) are obtained which is encouraging. The regression equation for basic nitrogen families is given in the Eq. (3):

3 $$\begin{aligned} \left[ {{\text{GC}} \times {\text{GC - NCD}}} \right]_{{{\text{predicted}}}} & =\upbeta _{{{\text{THQ}}\;{\text{Ani}}\;{\text{Pyr}}}} \times \left[ {{\text{FT - ICR}}\;{\text{MS}}} \right]_{{{\text{THQ}}\;{\text{Ani}}\;{\text{Pyr}}}} +\upbeta _{{{\text{Quinolines}}}} \\ & \quad \times \, \left[ {{\text{FT - ICR}}\;{\text{MS}}} \right]_{{{\text{Quinolines}}}} +\upbeta _{{{\text{Acridines}}}} \times \, \left[ {{\text{FT - ICR}}\;{\text{MS}}} \right]_{{{\text{Acridines}}}} \\ \end{aligned}$$

With here β_THQ Ani Pyr_ =  + 0.2179, β_Quinolines_ =  + 0.4932 and β_Acridines_ =  + 0.3145.

It is quite surprising that the quinolines and acridines pseudo-concentrations strongly influence the model and even in a higher extent than the THQ anilines pyridines pseudo-concentrations itself. The signs from each coefficient are positive indicating first that the THQ anilines pyridines family is globally underestimated when performing FT-ICR MS measurements, and second that the quinolines and acridines have a compensative action over the prediction model. This reveals that ionization competition phenomena are also taking place within basic nitrogen compounds and seem to be even more intense than those observed for neutral nitrogen compounds. Thus, the underestimation of THQ anilines pyridines pseudo-concentrations would be enhanced within samples that have high contents of quinolines and acridines. During positive electrospray ionization, the most basic species are more likely to be protonated and thus ionized. The pKb values of our compounds of interest are the following: THQ (7.7) < acridine (8.4) < pyridine (8.8) < quinoline (9.0) < anilines (9.4). Compounds with high pKb values have been shown to exhibit lower ESI(+) response^26^. The considered species here are not strong bases so that their full protonation in solution under working pH (≈ 2.5 after addition of 0.5% of acetic acid) is not likely to happen and their proton affinities in gas phase might better explain their ionization response. Gurinov et al.^27^. estimated the proton affinities of some of these compounds using DFT calculations. Under these simulated conditions, the proton affinities of the compounds are ranked as follows: pyridine (936 kJ/mol) < quinoline (963 kJ/mol) < tetrahydroquinoline (976 kJ/mol) < acridine (992 kJ/mol). Another study by Pankratov et al.^28^. suggested that the proton affinity of aniline is equal to 892 kJ/mol with a pKb equal to 9.4. The compounds that have the highest proton affinity should be more easily ionized^29^. However, another study from Ehrmann et al.^30^ showed that the predominant factor in the ionization of these basic molecules was their pKb in solution rather than their proton affinity. Thus, it was chosen here to try to discuss the relative ionization efficiencies of the compounds as a function of their pKb. As the THQ Anilines Pyridines family is a mix of three types of compounds that have different pKb values, the rationalization of its global ionization is quite tricky. However, based on the initial GC × GC-NCD blobs that allow the distinction and quantification of the THQ, anilines and pyridines compounds presented in Figure S1 in Supporting Information, it can be mentioned that the quantified amount of THQ in the samples is much lower than the amounts of anilines and pyridines so that the global behavior of the THQ anilines pyridines family should be mostly impacted by the pyridines and by the anilines in a lower extent. The pKb of aniline and pyridine compounds are higher than those observed for THQ and acridine and close to the one observed for quinoline (which could induce a strong competition between both compounds). This indicates that the THQ anilines pyridines family would be more likely to be underestimated when performing ESI hence enhancing the ionization of quinolines and acridines.

#### Quinolines family and their derivatives

As regards the quinolines family, the model was obtained excluding the GO 4 and GO 6. All predicted points are contained within the confidence intervals and near to the parity line as shown in Fig. 4. The determination coefficients are equal to 0.991 for calibration and 0.986 for cross validation which are very good figures of merit. The performances of this model were satisfying as the RMSEC and RMSECV values are respectively equal to 15 ppm and 20 ppm. The corresponding obtained regression coefficients are:

$$\upbeta _{{{\text{THQ}}\;{\text{Ani}}\;{\text{Pyr}}}} = + 0.6516,\;\upbeta _{{{\text{Quinolines}}}} = + 0.1932\;\;{\text{and}}\;\;\upbeta {\text{A}}_{{{\text{cridines}}}} = + 0.1920.$$

Again, the pseudo-concentrations from the three different families are responsible for the obtained prediction and especially the ones related to the THQ anilines pyridines family. The quinolines are then underestimated throughout FT-ICR MS analysis which is by the way clearly visible in Fig. 3. Compensative factors are then introduced thanks to the positive contributions from THQ anilines pyridines and acridines families so that the underestimation of the quinolines family would be enhanced for samples that have high contents of THQ anilines pyridines and acridines. The pKb of quinolines is higher than those observed for acridines which could party explain this global underestimation and also the contribution of acridines. As regards the contribution of THQ anilines pyridines family, it has been mentioned before that a strong ionization competition could take place between quinolines and pyridines as they have really close pKb.

#### Acridines family and their derivatives

Finally, the last model obtained for the acridines family excluded the GO 1 and GO 4 samples. Most of the samples are again contained within the confidence intervals. The determination coefficients of calibration (R^2^ = 0.947) and cross validation (R^2^ = 0.903) are quite satisfying. Low RMSEC (10 ppm) and RMSECV (14 ppm) are obtained indicating good accuracy of the model. The corresponding regression coefficients are the following:

$$\upbeta _{{{\text{THQ}}\;{\text{Ani}}\;{\text{Pyr}}}} = - 0.7379,\;\upbeta _{{{\text{Quinolines}}}} = + 1.92\;\;{\text{and}}\;\;\upbeta _{{{\text{Acridines}}}} = - 0.3329.$$

The three basic nitrogen families contribute to the model and one can note again the strong influence of the quinolines family. The signs related to the acridines and THQ anilines pyridines families are negative while the sign associated to the quinolines family is positive. This implies that the acridines should be globally overestimated in comparison to the observed GC × GC-NCD concentrations. The overestimation is enhanced within samples that have high contents of THQ anilines pyridines and on the opposite balanced within samples that have high contents in quinolines. The pKb of acridines is lower than most of the considered compounds (anilines, pyridines and quinolines) which could explain its favored ionization among other compounds. In particular, the pKb of pyridines and anilines compounds are lower than those of acridines which explains the enhanced overestimation of acridines in samples within high contents in THQ anilines pyridines are identified by GC × GC-NCD (hence explaining the negative coefficient of this family). As an example, the acridines pseudo-concentrations from sample GO 13 (classified as “other” with coordinates (152 ppm; 121 ppm) in Fig. 3) is over estimated and this gas oils reveals a high content in THQ anilines pyridines. On the opposite, the contribution of the quinolines is positive so that the overionization of the acridines family would be less important if a high amount of quinolines is found in the sample. Even, an underestimation of the acridines family could be suspected within samples with high quinolines contents such as GOCK samples as one can see from Fig. 3. This is quite surprising and could be linked to the alkylation degree of the samples: as an example, acridines in SRGO samples are globally underestimated (see Fig. 3) while they are overestimated in LCO samples. The acridines are globally more alkylated than quinolines in LCO samples whereas quinolines are slightly more alkylated than acridines in SRGO samples (see Figure S4 in Supporting Information). The pKb of an alkylated species is lower than those observed for the non-alkylated species and its proton affinity is higher^30^. Thus, the more alkylated character of the quinolines in GOCK samples would enhance their ionization response compared to the poorly alkylated acridines despite their lower pKb. To fully evaluate the influence of alkylation, a proper study should be performed on model molecules with different alkyl lengths which is beyond the scope of this work.

## Conclusion

The main goal of this paper was to evaluate the potential of ESI(+/−)-FT-ICR MS technique as a pseudo-quantitative tool for the characterization of nitrogen compounds in gas oils in a non-targeted direct infusion approach using both GC × GC and MLR tools. It is known that the electrospray ionization is complex and unrationalized due to different factors. Among them, we can mention the matrix effect, the differences in ionization efficiencies due to the structures/composition of the molecules inducing ionization competitions or again the ionization conditions. To evaluate the extent of some of these phenomenon, the concentrations obtained by the reference method GC × GC-NCD for the main nitrogen families were compared to the pseudo-concentrations obtained by FT-ICR MS. To do this, the reference GC × GC-NCD method has been first optimized using GC × GC/HRMS to verify the chemical formula assignments and create some identification blobs as a function of the DBE values of the compounds so that they can be directly compared to FT-ICR MS measurements. The direct comparison of FT-ICR MS and GC × GC-NCD has showed that there is no linear trend between both techniques when considering very different types of gas oils and so that the ionization response of the nitrogen compounds is also depending on the intrinsic gas oil matrix. It was therefore not possible to correct the FT-ICR MS values directly by a factor to make them quantitative. As a consequence, multiple linear regression models have been developed for each nitrogen family to predict the GC × GC-NCD concentrations of the gas oils considering not only FT-ICR MS measurements for the given family but also FT-ICR MS measurements from other nitrogen families. The benefits from this approach were multiple. First, some linear trends were observed between the measured and the predicted values indicating that satisfying predictive models have been developed. Second, the addition of the other nitrogen families to the predictive model allowed correcting both the intrinsic matrix effect linked to the type of gas oil and the differences in ionization responses to obtain a common mean ionization response for all gas oils. Last, the study of the regression coefficients for each model has allowed to start rationalizing the ionization response of the nitrogen compounds depending on either a corrective or a compensative factor was applied. Some differences have been spotted between the different families as well as within the different types of gas oils highlighting the very complex ionization competition taking place. The suggested ionization competitions between nitrogen compounds are only one of the many factors impacting the ionization of compounds as the structures of the molecules (isomers) are also a key parameter as well as the polarity of the samples that could also be assessed through an hyphenated approach with reversed-phase HPLC. Also, the effects of the alkylation and the solvation of the molecule over the ionization response have not been studied in this paper and should be considered within future studies. This approach has been used here for nitrogen compounds within gas oils but is promising for other studies focusing on the implementation of a pseudo-quantitative analysis of complex matrices using ESI-FT-ICR MS.

## Methods

### Gas oil samples and pre-fractionation

21 different gas oils with various industrial origins were analyzed in this study to evaluate the gas oil matrix effect over the ionization of nitrogen compounds. The macroscopic properties of these samples are shown in Table 1.

A set of representative gas oils, GO 3 (Straight Run, SRGO), GO 5 (Light Cycle Oil, LCO), GO 10 (Coker Gas Oil, GOCK), GO 12 (Ebullated-Bed Gas Oil, EBGO) and GO 17 (Hydrotreated Gas Oil, HDT), has been pre-fractionated to remove the hydrocarbon matrix prior to GC × GC/HRMS analysis using a Flash HPLC 2250 chromatograph (Gilson, WI, USA) following the protocol developed by Proriol et al.^25^. 5 fractions have been obtained and evaporated using gentle nitrogen bath (about 1 bar) without heating to prevent the evaporation of light compounds.

### FT-ICR MS analysis

The 21 gas oils were analyzed by ESI(+/−)-FT-ICR MS considering 6 technical replicates using a LTQ FT Ultra Fourier Transform Ion Cyclotron Resonance Mass Spectrometer (FT-ICR MS) (ThermoFisher Scientific, Bremen Germany) equipped with a 7 T magnet (Oxford Instruments) and hyphenated with an ESI source (ThermoFisher Scientific) used in positive and negative modes. The chosen mass range was equal to m/z 98–1000. 70 scans with 4 µ-scans and their corresponding transients were recorded with an initial resolution set to 200,000 at m/z 300 before data processing. The ionization and ion transfer conditions for each ionization mode are described elsewhere^16^. The external mass calibration was performed using a sodium formate clusters solution (sodium formiate provided from VWR, Fontenay-sous-Bois, France) from about 90 to 1000 Da.

The full data processing has been described elsewhere^16^. Phase absorption and phase correction were applied on a summed transient (sum of 70 recorded transients). Using this approach, the resolution was then equal to 700,000 at m/z 300 and the mass error was lower than 50 ppb. The corresponding mass spectrum was then noise thresholded and peak picked by a home-made software to assign the molecular formula with the following conditions: C0-50H0-100O0-3N0-3S0-3. The maximum content of heteroatoms in one molecular formula was set to 3 and 1 ppm of maximum mass error after mass recalibration based on N1[H] peaks. As GC × GC-NCD quantifies N1-type compounds, only N1[H] peaks were considered here and their relative intensities have been calculated as follows: the N1[H] peak intensity divided by the sum of intensities from all N1[H] peaks. The pseudo-concentrations were then obtained by multiplying the relative intensity times the amount of elemental neutral nitrogen (for ESI(-) data) or basic nitrogen (for ESI(+) data).

### GC × GC-NCD analysis

GC × GC-NCD analyses were performed on gas oils with an Agilent 7890A gas chromatograph equipped with a 5%diphenyl-95%dimethylsiloxane non-polar column (SPB5, Sigma-Aldrich, 27.5 m × 0.25 mm × 1 µm) and a second polar column composed of polyethylene glycol (SolgelWax, SGE, 1.5 m × 0.1 mm × 0.1 µm). An on-column injector operated at 260 °C was used. The injection volume was set to 0.5 µL. A constant flow rate of 1.4 mL/min of helium gas was held during all analysis. Oven temperature was initially set to 60 °C and increased to 260 °C at a rate of 3 °C/min. The NCD Detector was a 255 Dual Plasma from Sievers (Agilent, Santa Clara, CA, USA) operated at 1000 °C. Modulation period was 12 s with a 0.6 s hot jet and 2.5% mass cold flow. Data were processed with a home-made software called 2D Chrom for contour plotting, retention time measurement, blobs fitting and blobs quantitation. The concentrations were obtained by multiplying the relative area of the families times the total amount of nitrogen in the sample.

### GC × GC/HRMS analysis

GC × GC/HRMS analyses were performed on pre-fractionated gas oils with an Agilent 7890A gas chromatograph hyphenated to a LECO Pegasus 4D-HRT+ (LECO, St Joseph, MI, USA) used in high resolution mode equipped with an electron ionization source (70 eV). The same columns and chromatographic conditions previously described for GC × GC-NCD analysis were used here to compare both chromatograms for given gas oils. However, a split injector was used for GC × GC/HRMS instead of an on-column injector with a split ratio equal to 50. The ion source temperature was kept at 280 °C as well as the transfer line for all measurements. The m/z range selected was 30–550 Da. The detector voltage was 2050 V and the acquisition frequency was 200 spectra per second. Perfluorotributylamine (PFTBA) was added to the gas phase as internal standard for mass calibration at the end of the analysis to perform mass recalibration. The LECO software (ChromaTOF version 5.2) was used for data processing.

### Multiple linear regression

A multiple linear regression is a statistical tool used to identify a potential linear combination of p input variables xi in order to predict one output variable y using a least-squares optimization (Eq. 4):

4 $$y = \beta_{0} + \beta_{1} x_{1} + \beta_{2} x_{2} + \cdots + \beta_{i} x_{i} + \cdots + \beta_{p} x_{p}$$

On the basis of n known samples, the calibration step therefore consists of finding the optimal beta coefficients making the link between input and output variables. In a natural way, each of the n samples in the considered data set must follow this equation which allows us to obtain a system of n equations that we have to solve:A matrix notation thus makes it possible to write: with x_j,i_ the input value of the parameter *i* for the sample *j* and y_j_ the output value of the same sample.

$$\left[ {\begin{array}{*{20}c} {y_{1} } \\ {y_{2} } \\ \ldots \\ {y_{j} } \\ \ldots \\ {y_{n} } \\ \end{array} } \right] = \left[ {\begin{array}{*{20}c} {x_{1,1} } & {\quad x_{1,2} } & {\quad \ldots } & {\quad x_{1,i} } & {\quad \ldots } & {\quad x_{1,p} } \\ {x_{2,1} } & {\quad x_{2,2} } & {\quad \ldots } & {\quad x_{2,i} } & {\quad \ldots } & {\quad x_{2,p} } \\ \ldots & {\quad \ldots } & {\quad \ldots } & {\quad \ldots } & {\quad \ldots } & {\quad \ldots } \\ {x_{j,1} } & {\quad x_{j,2} } & {\quad \ldots } & {\quad x_{j,i} } & {\quad \ldots } & {\quad x_{j,p} } \\ \ldots & {\quad \ldots } & {\quad \ldots } & {\quad \ldots } & {\quad \ldots } & {\quad \ldots } \\ {x_{n,1} } & { \quad x_{n,2} } & {\quad \ldots } & { \quad x_{n,i} } & {\quad \ldots } & {\quad x_{n,p} } \\ \end{array} } \right]\left[ {\begin{array}{*{20}c} {\beta_{1} } \\ {\beta_{2} } \\ \ldots \\ {\beta_{i} } \\ \ldots \\ {\beta_{p} } \\ \end{array} } \right].\;{\text{or}}\;{\text{y}} = {\text{X}}\upbeta$$

During the calibration step, all elements of the y and X matrices are known and the least-squares optimization allows us obtaining all the elements of the β matrix which are the coefficients of regression. Thus in the proposed work, FT-ICR MS measurements from different nitrogen families (called input variables) will be used to predict the concentration we could obtain using GC × GC-NCD for various classes of molecules. More specifically, for each nitrogen family, the y matrix (dimension 21 × 1) will contain the GC × GC-NCD concentrations of the 21 gas oils samples and the X matrix will contain the corresponding FT-ICR MS pseudo-concentrations of the indoles and the carbazoles families for neutral nitrogen families (dimension 21 × 2), and the FT-ICR MS pseudo-concentrations of the THQ anilines pyridines, quinolines and acridines for basic nitrogen families (dimension 21 × 3). Since all the variables have potentially very different variances, we will apply the so-called autoscale preprocessing to give each of them a chance to express themselves in this predictive model. In fact, at the end of this preprocessing step, each variable will have the same mean and standard deviation, i.e. 0 and 1 respectively. Thanks to this, we will be able to directly use the values of the coefficients of a model in order to apprehend the effects of each variable and their relative importance for the prediction of concentrations. The efficiency of a model to predict a chosen family is estimated considering different figures of merit such as the Root Mean Square Error of Calibration (RMSEC) which should be as low as possible indicating small differences between predicted concentration values and reference ones. However, only a validation step will allow us to estimate the real accuracy of the constructed models. As a consequence, a venitian-blind cross validation will be performed for each model and the corresponding Root Mean Square Error of Cross Validation (RMSECV) will be evaluated. All MLR models were developed using the PLS_Toolbox (Eigenvector Research Inc, Wenatchee, WA, USA) version 8.6 for MATLAB (R2018B).

## Supplementary Information

Supplementary Figures and Tables

## References

[1] von Mühlen C, de Oliveira EC, Zini CA, Caramão EB, Marriott PJ (2010). Characterization of nitrogen-containing compounds in heavy gas oil petroleum fractions using comprehensive two-dimensional gas chromatography coupled to time-of-flight mass spectrometry. Energy Fuels.

[2] Flego C, Zannoni C (2011). N-containing species in crude oil fractions: an identification and quantification method by comprehensive two-dimensional gas chromatography coupled with quadrupole mass spectrometry. Fuel.

[3] Yan X (2006). Unique selective detectors for gas chromatography: nitrogen and sulfur chemiluminescence detectors. J. Sep. Sci..

[4] Adam F, Bertoncini F, Brodusch N, Durand E, Thiébaut D, Espinat D, Hennion M-C (2007). New benchmark for basic and neutral nitrogen compounds speciation in middle distillates using comprehensive two-dimensional gas chromatography. J. Chromatogr. A.

[5] Chawla B (1997). Speciation of nitrogen compounds in gasoline and diesel range process streams by capillary column gas chromatography with chemiluminescence detection. J. Chromatogr. Sci..

[6] Lorentz C, Laurenti D, Zotin JL, Geantet C (2017). Comprehensive GC × GC chromatography for the characterization of sulfur compound in fuels: a review. Catal. Today.

[7] Marshall AG, Rodgers RP (2004). Petroleomics: the next grand challenge for chemical analysis. Acc. Chem. Res..

[8] Marshall AG, Rodgers RP (2008). Petroleomics: chemistry of the underworld. PNAS.

[9] Ruddy BM, Hendrickson CL, Rodgers RP, Marshall AG (2018). Positive Ion electrospray ionization suppression in petroleum and complex mixtures. Energy Fuels.

[10] Barbosa LL, Sad CMS, Morgan VG, Figueiras PR, Castro ERV (2016). Application of low field NMR as an alternative technique to quantification of total acid number and sulphur content in petroleum from Brazilian reservoirs. Fuel.

[11] Molina D, Uribe UN, Murgich J (2010). Correlations between SARA fractions and physicochemical properties with 1H NMR spectra of vacuum residues from Colombian crude oils. Fuel.

[12] Cakara A, Bonta M, Riedl H, Mayrhofer PH, Limbeck A (2016). Development of a multi-variate calibration approach for quantitative analysis of oxidation resistant Mo–Si–B coatings using laser ablation inductively coupled plasma mass spectrometry. Spectrochim. Acta Part B.

[13] Sau M, Basak K, Manna U, Santra M, Verma RP (2005). Effects of organic nitrogen compounds on hydrotreating and hydrocracking reactions. Catal. Today.

[14] Le Maître J, Hubert-Roux M, Paupy B, Marceau S, Rüger CP, Afonso C, Giusti P (2019). Structural analysis of heavy oil fractions after hydrodenitrogenation by high-resolution tandem mass spectrometry and ion mobility spectrometry. Faraday Discuss..

[15] Nguyen MT, Pirngruber G, Chainet F, Albrieux F, Tayakout-Fayolle M, Geantet C (2019). Molecular level insights into straight run/Coker gas oil hydrodenitrogenation by Fourier transform ion cyclotron resonance mass spectrometry. Energy Fuels.

[16] Guillemant J, Albrieux F, de Oliveira LP, Lacoue-Nègre M, Duponchel L, Joly J-F (2019). Insights from nitrogen compounds in gas oils highlighted by high-resolution Fourier transform mass spectrometry. Anal. Chem..

[17] Rabarihoela-Rakotovao V, Diehl F, Brunet S (2009). Deep HDS of diesel fuel: inhibiting effect of nitrogen compounds on the transformation of the refractory 4,6-dimethyldibenzothiophene over a NiMoP/Al_2_O_3_ catalyst. Catal Lett.

[18] Shin S, Sakanishi K, Mochida I, Grudoski DA, Shinn JH (2000). Identification and reactivity of nitrogen molecular species in gas oils. Energy Fuels.

[19] López García C, Hudebine D, Schweitzer J-M, Verstraete JJ, Ferré D (2010). In-depth modeling of gas oil hydrotreating: from feedstock reconstruction to reactor stability analysis. Catal. Today.

[20] Hughey CA, Hendrickson CL, Rodgers RP, Marshall AG (2001). Elemental composition analysis of processed and unprocessed diesel fuel by electrospray ionization Fourier transform ion cyclotron resonance mass spectrometry. Energy Fuels.

[21] Chen X, Shen B, Sun J, Wang C, Shan H, Yang C, Li C (2012). Characterization and comparison of nitrogen compounds in hydrotreated and untreated shale oil by electrospray ionization (ESI) Fourier transform ion cyclotron resonance mass spectrometry (FT-ICR MS). Energy Fuels.

[22] Adam F, Muller H, Al-Hajji A, Bourane A, Koseoglu O (2015). Oxidative desulfurization process monitoring using comprehensive two-dimensional gas chromatography and Fourier transform ion cyclotron resonance mass spectrometry. Energy Fuels.

[23] Dutriez T, Courtiade M, Ponthus J, Thiébaut D, Dulot H, Hennion M-C (2012). Complementarity of Fourier transform ion cyclotron resonance mass spectrometry and high temperature comprehensive two-dimensional gas chromatography for the characterization of resin fractions from vacuum gas oils. Fuel.

[24] Muller H, Adam FM, Panda SK, Al-Jawad HH, Al-Hajji AA (2012). Evaluation of quantitative sulfur speciation in gas oils by Fourier transform ion cyclotron resonance mass spectrometry: validation by comprehensive two-dimensional gas chromatography. J. Am. Soc. Mass Spectrom..

[25] Proriol D, Chahen L (2020). Toward new descriptors of basic nitrogen compounds in middle distillates by 2D NMR. Energy Fuels.

[26] Hughey CA, Rodgers RP, Marshall AG, Qian K, Robbins WK (2002). Identification of acidic NSO compounds in crude oils of different geochemical origins by negative ion electrospray Fourier transform ion cyclotron resonance mass spectrometry. Org. Geochem..

[27] Gurinov AA, Denisov GS, Borissova AO, Goloveshkin AS, Greindl J, Limbach H-H, Shenderovich IG (2017). NMR study of solvation effect on the geometry of proton-bound homodimers of increasing size. J. Phys. Chem. A.

[28] Pankratov AN, Uchaeva IM, Yu S, Chernova D, Chenova RK (2001). Gaseous-phase proton affinity of anilines: a quantum chemical evaluation and discussion in view of aqueous basicity. J. Serb. Chem. Soc..

[29] Amad MAH, Cech NB, Jackson GS, Enke CG (2000). Importance of gas-phase proton affinities in determining the electrospray ionization response for analytes and solvents. J. Mass Spectrom..

[30] Ehrmann BM, Henriksen T, Cech NB (2008). Relative importance of basicity in the gas phase and in solution for determining selectivity in electrospray ionization mass spectrometry. J. Am. Soc. Mass Spectrom..

