# Supplementary material for: Towards a new pseudo-quantitative approach to evaluate the ionization response of nitrogen compounds in complex matrices
Source: Sci Rep. 2021 Mar 19;11:6417. doi: 10.1038/s41598-021-85854-7 (PMC7979777; doi:10.1038/s41598-021-85854-7)
Supplement: Supplementary file 1 — Supplementary Figures and Tables [file 41598_2021_85854_MOESM1_ESM.docx]

Supporting Information

**Towards a new pseudo-quantitative approach to evaluate the ionization response of nitrogen compounds in complex matrices**

Julie Guillemant†, Marion Lacoue-Nègre†, Alexandra Berlioz-Barbier*†, Florian Albrieux†, Luis P. de Oliveira†, Jean-François Joly†, and Ludovic Duponchel*‡

† IFP Energies nouvelles, Rond-point de l'échangeur de Solaize, BP 3, 69360 Solaize, France

‡ Univ. Lille, CNRS, UMR 8516 - LASIR – Laboratoire de Spectrochimie Infrarouge et Raman, F-59000 Lille, France

* Correspondence should be addressed to Dr. Alexandra Berlioz-Barbier or Prof. Ludovic Duponchel: alexandra.berlioz-barbier@ifpen.fr or ludovic.duponchel@univ-lille.fr

**Table of contents**

| **Figure/Table** | **Reference** | **Page** |
| --- | --- | --- |
| Initial GC×GC-NCD identification blobs | Figure S1 | S-2 |
| Comparison of the F3F4F5 solutions and whole gas oils analysis in ESI(+) mode | Figure S2 | S-3 |
| Comparison of the F3F4F5 solutions and whole gas oils analysis in ESI(-) mode | Figure S3 | S-4 |
| Mass spectra and DBE=f(#C) plots in ESI(+) mode | Figure S4 | S-5 |
| Mass spectra and DBE=f(#C) plots in ESI(-) mode | Figure S5 | S-6 |
| Characteristics of MLR models | Table S1 | S-7 |
| Comparison of the alkylation of quinolines and acridines families within samples GO 3 and GO 5 | Figure S6 | S-8 |

Figure S1: Initial GC×GC-NCD identification blobs


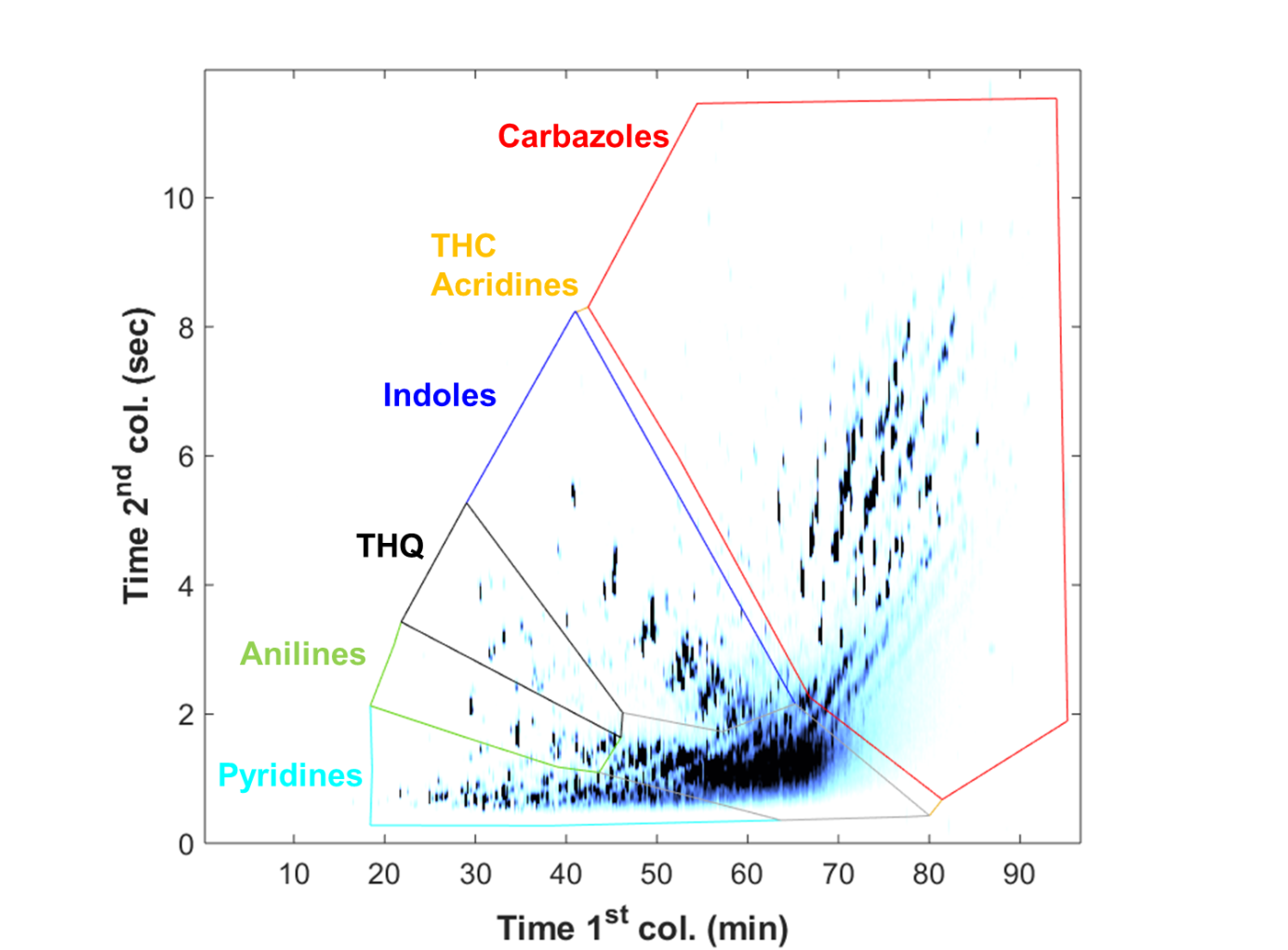


Figure S2: Comparison of the F3F4F5 solutions and whole gas oils analysis in ESI(+) mode


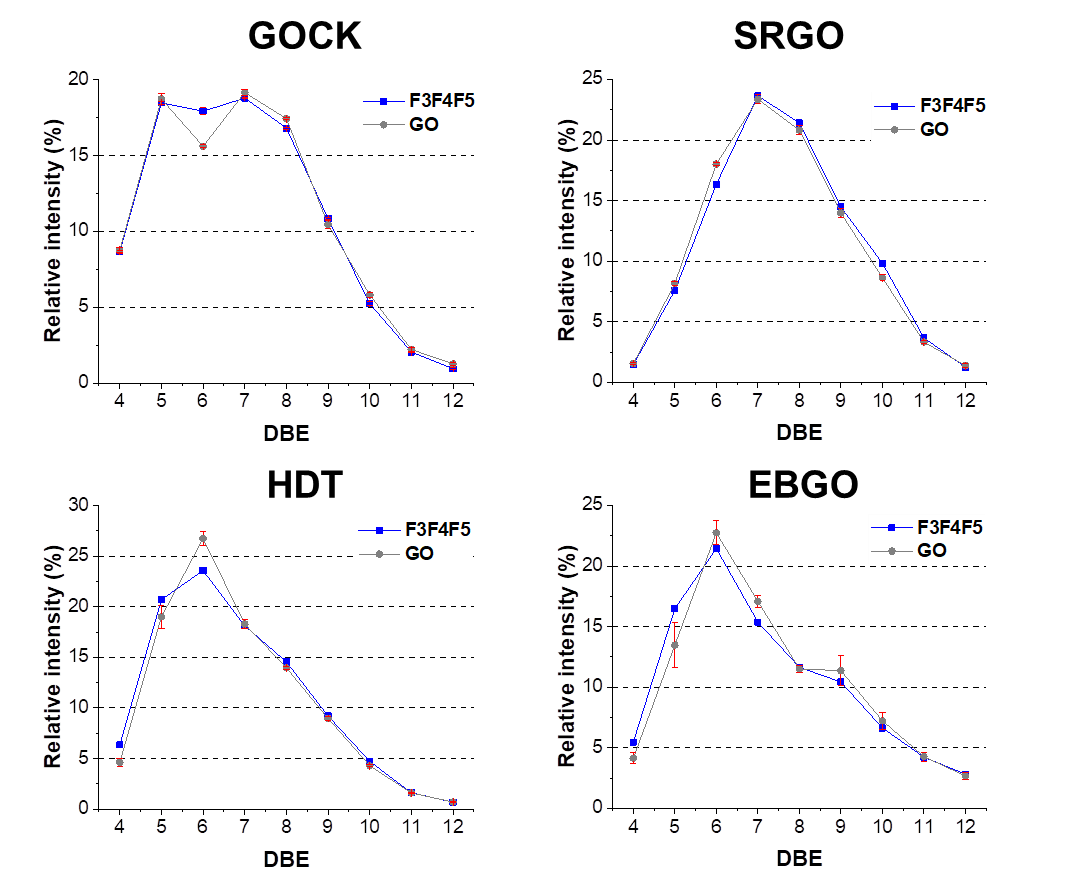


**Figure S3: Comparison of the F3F4F5 solutions and whole gas oils analysis in ESI(-) mode**


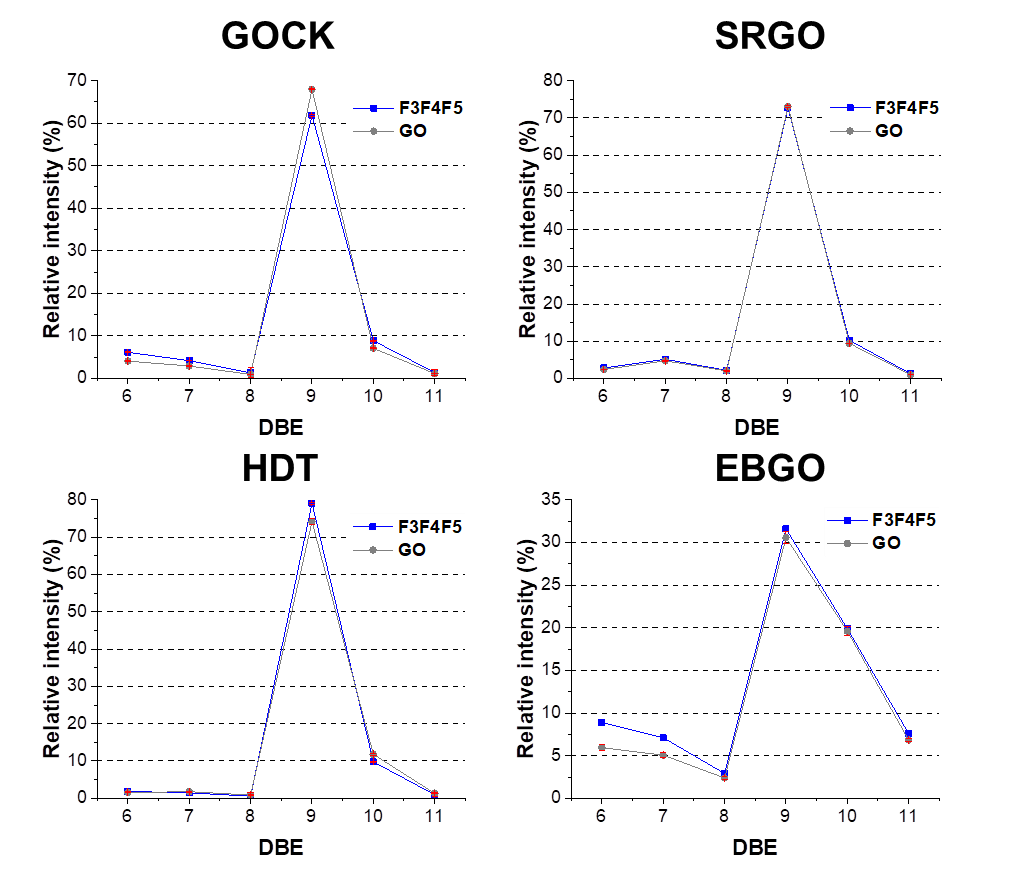


**Figure S4: (A) Mass spectra obtained and (B) their corresponding DBE=f(#C) plots for the N1[H] class in ESI(+) mode for 4 types of different gas oils. (C) Number of N1[H] compounds identified depending on the gas oil considered.**


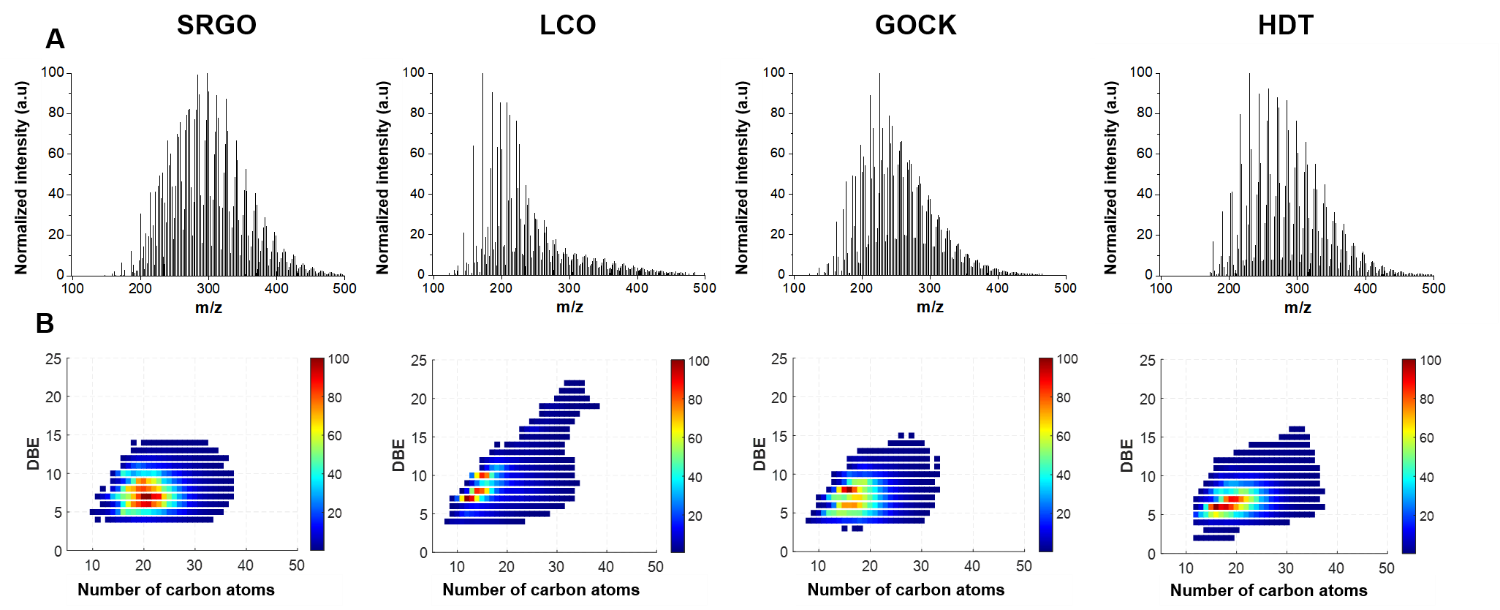


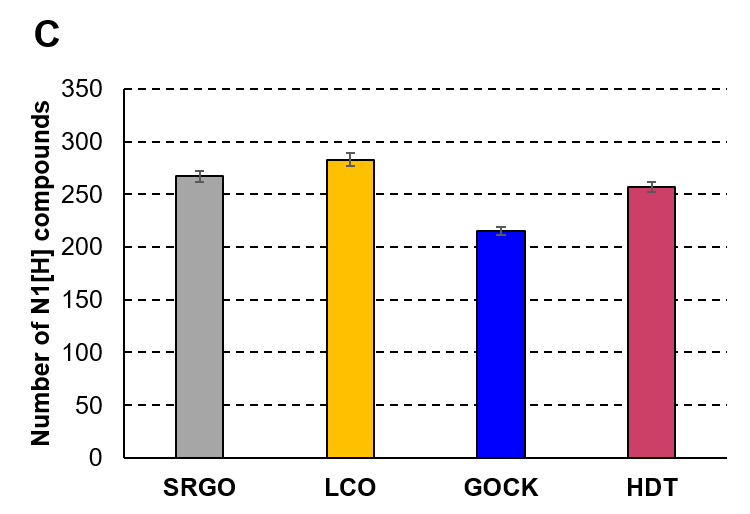


**Figure S5: (A) Mass spectra obtained and (B) their corresponding DBE=f(#C) plots for the N1[H] class in ESI(-) mode for 4 types of different gas oils. (C) Number of N1[H] compounds identified depending on the gas oil considered.**


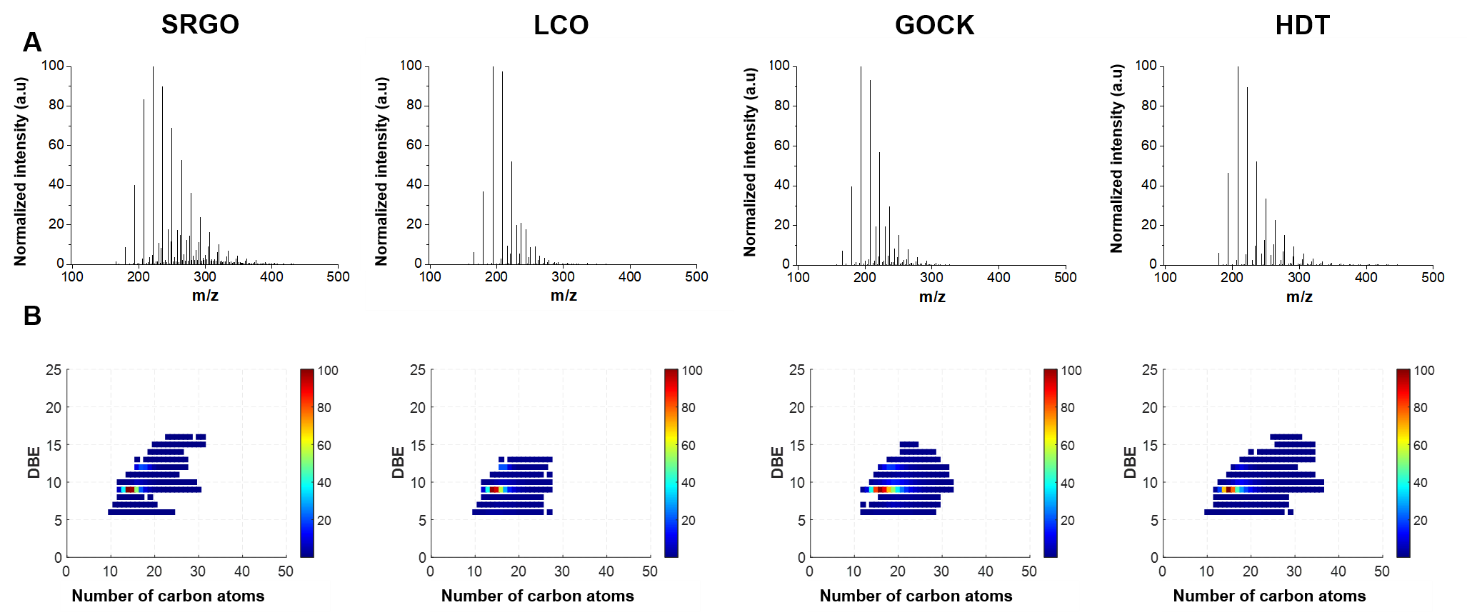

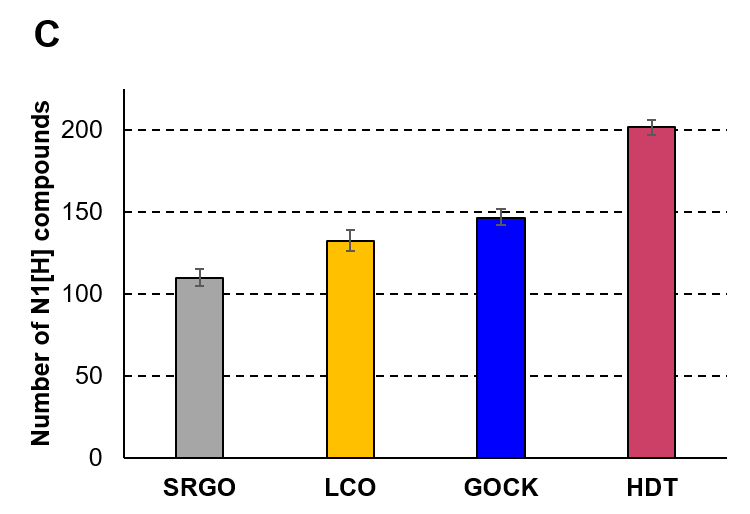


Table S1. Characteristics of MLR models. *: the reference error of the GC×GC-NCD method was assumed to be equal to 10%.

| **Criteria** | **Indoles** | **Carbazoles** | **THQ Ani Pyr** | **Quinolines** | **Acridines** |
| --- | --- | --- | --- | --- | --- |
| Outliers | GO 1, GO 7, GO 21 | GO 4, GO 6, GO 18 | GO 6, GO 11, GO 18 | GO 4, GO 6 | GO 1, GO 4 |
| RMSEC (ppm) | 19 | 57 | 18 | 15 | 10 |
| RMSECV (ppm) | 20 | 70 | 23 | 20 | 14 |
| Concentration range (ppm) | 0-270 | 0-1000 | 0-400 | 0-650 | 0-170 |
| Robustness criteria (RMSECV/RMSEC) | 1.05 | 1.22 | 1.32 | 1.26 | 1.35 |
| Cross validation bias (%) | 0.047 | 0.511 | 0.159 | -0.263 | -0.640 |
| Bias | 0 | 1.14E-13 | 1.42E-14 | 2.84E-14 | 2.13E-14 |
| R²_calibration_ | 0.939 | 0.932 | 0.965 | 0.991 | 0.947 |
| R²_cross validation_ | 0.932 | 0.898 | 0.938 | 0.986 | 0.902 |
| CV: Number of splits | 8 | 10 | 5 | 8 | 6 |
| CV: Samples per blind | 2 | 1 | 3 | 2 | 2 |
| GC×GC-NCD  Error (%) | 10* | 10* | 10* | 10* | 10* |

**Figure S6: Comparison of the evolution of the nitrogen pseudo-concentration as a function of number of carbon atoms for the quinolines and acridines families for the samples GO 3 (SRGO) and GO 5 (LCO)**

**
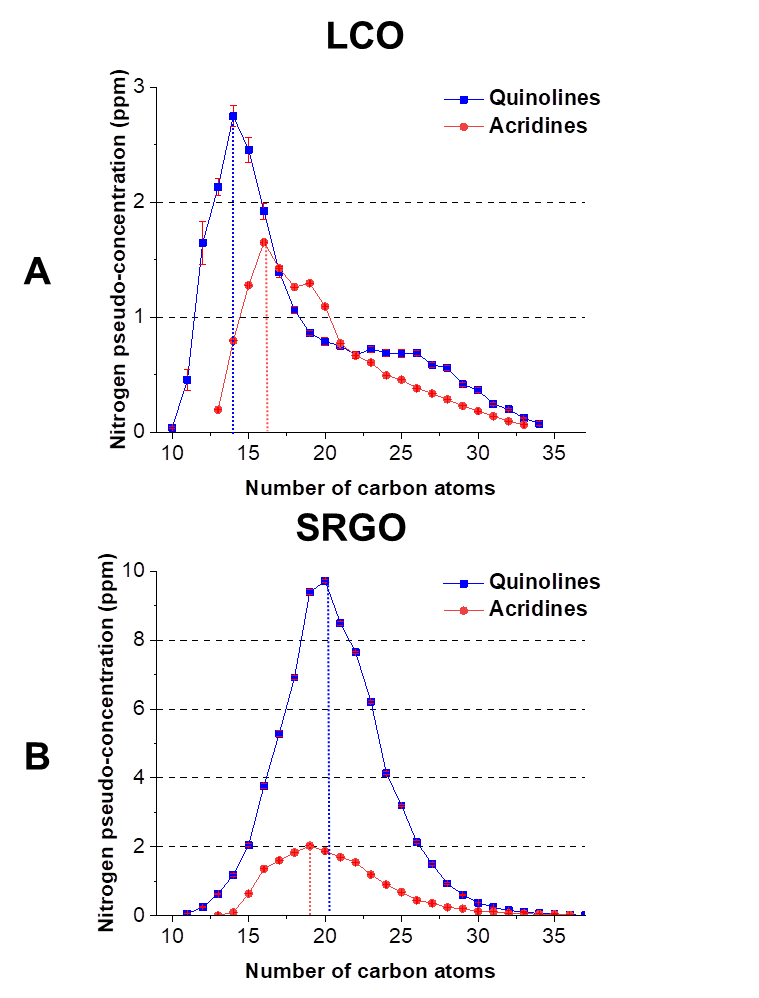
**
